# Having mentors and campus social networks moderates the impact of worries and video gaming on depressive symptoms: a moderated mediation analysis

**DOI:** 10.1186/1471-2458-14-426

**Published:** 2014-05-05

**Authors:** Jong-Sun Lee, Bumseok Jeong

**Affiliations:** 1 Laboratory of Clinical Neuroscience and Development, Graduate School of Medical Science and Engineering, KAIST, Daejeon, Republic of Korea

**Keywords:** Depression, Worry, Mentor, Social network, Young adult, Internet gaming

## Abstract

**Background:**

Easy access to the internet has spawned a wealth of research to investigate the effects of its use on depression. However, one limitation of many previous studies is that they disregard the interactive mechanisms of risk and protective factors. The aim of the present study was to investigate a resilience model in the relationship between worry, daily internet video game playing, daily sleep duration, mentors, social networks and depression, using a moderated mediation analysis.

**Methods:**

6068 Korean undergraduate and graduate students participated in this study. The participants completed a web-based mental health screening questionnaire including the Beck Depression Inventory (BDI) and information about number of worries, number of mentors, number of campus social networks, daily sleep duration, daily amount of internet video game playing and daily amount of internet searching on computer or smartphone. A moderated mediation analysis was carried out using the PROCESS macro which allowed the inclusion of mediators and moderator in the same model.

**Results:**

The results showed that the daily amount of internet video game playing and daily sleep duration partially mediated the association between the number of worries and the severity of depression. In addition, the mediating effect of the daily amount of internet video game playing was moderated by both the number of mentors and the number of campus social networks.

**Conclusions:**

The current findings indicate that the negative impact of worry on depression through internet video game playing can be buffered when students seek to have a number of mentors and campus social networks. Interventions should therefore target individuals who have higher number of worries but seek only a few mentors or campus social networks. Social support via campus mentorship and social networks ameliorate the severity of depression in university students.

## Background

Depression is a common psychiatric disorder, and it is predicted to be the second most debilitating disorder by 2020 [1]. While there has been increasing concern about depression in specific groups, such as adolescents or the elderly [2,3], depression in university students is also of great importance. Most life time mental disorders manifest during or shortly before the age at which people enter university [4], and depression rates reported by university students is steadily increasing [5], and are even higher than those of the general population [6]. Therefore, research on depression in university students has crucial implications. Moreover, if unidentified and untreated, depression in university students is a costly problem, in that its impact is far-reaching throughout adulthood, affecting areas such as future career development, interpersonal relationships, work performance, and further development of psychiatric disorders [7-9].

While the risk factors of childhood traumatic events [10,11], insecure attachment [12,13], maladaptive cognition [14], and substance abuse [15] for depression have been well researched, the role of daily worries on depression in university students has not been examined. University students are in a critical transition period during which they face many stressors such as an upsurge of social networks, academic pressure and financial struggles, which can increase the number of worries or concerns. If overwhelming, these factors along with past experiences, such as childhood trauma, may contribute to their depression [16]. Indeed, there has been growing evidence that worry is significantly associated with depression [17,18]. Such worries, especially family-, health- and school-related ones, tend to be the most common precipitating factors of insomnia [19,20]. Also, recent studies have found that short sleep duration is a key predictor of persistent insomnia which is known to be a significant risk factor for the development of depression [21,22]. These results suggest that short sleep duration might mediate the association between daily worries and depression.

Another potential factor associated with depression is excessive internet use on the computer or smartphone. However, the impact of its use on depression depends on the type of internet activity. For adolescents who reported low quality of friendships, non-communicative internet use such as simple surfing predicted an increased risk of depression whereas communicative internet use, such as instant messaging predicted less depression [23]. Similarly, hours per week used for email, chat room or instant messaging in freshmen college students was associated with lower levels of depressive symptoms, whereas hours per week for shopping, playing games, or research was related to a higher levels of depressive symptoms [24]. Another study also found that internet use for health purposes appeared to be associated with increased depression, whereas the internet use for communicative purposes with friends, family or others was related to a decrease in depression [25].

Among internet activities for non-communicative purposes, problematic video game playing/video game addiction, but not video game playing in general, appeared to be associated with negative mental health outcomes [26-28]. For example, the relationship between loneliness and pathological video gaming was reciprocal while lower psychosocial well-being, such as social incompetence and low self-esteem was an antecedent of pathological gaming [29]. A recent meta-analysis, however, showed that the prevalence of pathological gaming is overestimated, and comorbidity between pathological gaming and mental health ranges small to moderate, indicating a need for the development of a sensitive and specific diagnostic tool for video gaming [30]. On the other hand, the underlying mechanism of problematic video game playing might be a maladaptive coping response to other problems that people are escaping from [31]. If this is the case, it is likely that problematic video gaming plays a mediator role in the relationship between real life problems and mental health. However, there is yet to be a study examining the mediating effect of video games on mental health.

Recently, there has been a growing interest in investigating resilience factors on mental health. This is because not all university students with risk factors experience depression; some are able to overcome their difficulties and make health developments. However, it is surprising that research on resilience, especially for depression in university students, is largely scarce [32]. The protective (resilience) factors can be individual assets (e.g., coping skills, self-efficacy) or external resources (e.g., parental support, mentoring) that promote healthy development [33]. There has been evidence that self-efficacy or self-esteem plays a protective role against depressive symptoms [34-36]. Previous studies have also shown that social support from parents, friends, or mentors who provide emotional and practical support or who can be consulted when someone is suffering from a problems, significant influential social factors for depressive symptoms in adolescents and college students [24,37-40]. For example, a longitudinal study showed that perceived social support predicted lower levels of depression over a six month period in college students [41]. While the role of social support on depression has been emphasized so far, it is still unclear to what extent mentors or social networks would be needed to buffer the detrimental impact of daily worries and video gaming on depression in university students. In this sense, the number of mentors or social networks would provide clear and useful information. Surprisingly, there is yet to be a study to examine the number of mentors or social networks as a possible moderator in university students.

Resilience models consider not only risk factors but also protective factors that interact to diminish negative outcomes and promotes positive ones [33]. As such, the present study aimed to investigate the interaction of the risks and protective factors related to depression in the same model, using a moderated mediation analysis. Moderated mediation analysis was recently proposed by Preacher and others [42,43], it offers a much improved model estimation over previous methods since it performs mediation and moderation together in the same model. In particular, a recent PROCESS macro identifies the ranges of the moderators (e.g., Johnson-Neyman technique), for which a conditional indirect effect is statistically significant, and it allows the inclusion of multiple mediators [43].

**Figure 1:**
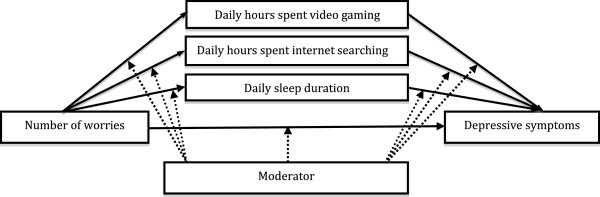
**Proposed moderated mediation model.** The model was attested with moderators such as mentors, campus social networks, or both using the first eigenvalue.

Considered collectively, based on the above evidence, the present study began with the following hypotheses: 1) daily worries would increase the risk of developing depression through decreased daily sleep duration, excessive internet searching and video game playing, 2) some students who have a higher number of mentors or social networks would be protected from such negative impact of daily worries on depression by having a longer daily sleep duration and less internet searching and video gaming, compared to have a lower number of mentors or social networks. To examine the aforementioned hypotheses, firstly, it was tested whether the daily hours spent playing video games, the daily amount of internet searching on a computer or smartphone, or daily sleep duration would mediate the relationship between the number of worries and depression (mediation). Secondly, we tested whether the indirect effect of the number of worries on depression via the daily amount of video game playing, daily amount of internet searching, and daily sleep duration would be moderated by the number of mentors or campus social networks a student has, respectively (moderated mediation). This study is the first to investigate the interaction between risks and protective factors in depression in university students by applying a moderated mediation model. Figure 1 depicts proposed moderated mediation model in the present study.

## Methods

### Participants and procedure

The participants were undergraduate and graduate students who were enrolled at a national university, Daejeon, Republic of Korea. 6173 students (undergraduate: 2995; graduate: 3178) out of 10531 students (undergraduate: 4822; graduate: 5709) completed a web-based mental health questionnaire with informed consent. The participants were informed that the purpose of the study was to investigate the relationship between their interpersonal relationships and mental health. However, 49 students were excluded as a result of missing data, and 2 students were excluded since they were identified as statistical outliers. The final analysis included 6068 students; 48.2% (*N* = 2927) were undergraduate students and 51.8% (*N* = 3141) were graduate students. Female students made up 23.9% (*N* = 1450) of the sample and male students 76.1% (N = 4618). Ages ranged from 18 to 30 years old (*M* = 20.29 ± 1.72) for undergraduate students and from 20 to 47 years old (*M* = 26.43 ± 3.19) for graduate students. The study was approved by the Institutional Review Board at the Korean Advanced Institute of Science and Technology in Daejeon, Republic of Korea (KH2012-16). The students, aged 18 to 19 years old, were judged as independent in taking part in the present research; therefore the written consent form was completed by them rather than their parent/guardian/carer. This procedure was approved by the Institutional Review Board at the Korean Advanced Institute of Science and Technology in Daejeon, Republic of Korea (KH2012-16).

### Measures

The severity of depressive symptoms was measured using the validated Korean version of the Beck Depression Inventory [44]. The BDI is a “multiple-choice, self-report, inventory” consisting of 21 items [45]. Total scores range from 0 to 63. Higher total scores are indicative of more severe levels of depression. Previous research on the BDI has reported a good Cronbach’s alpha coefficient of 0.81 for non-psychiatric populations and of 0.86 for psychiatric patients [45]. The validated Korean version of the BDI showed good psychometric properties: Cronbach’s α = 0.93, test-retest reliability coefficient r = 0.91 and consistency coefficient = 0.85 [44].

Information about the number of worries was collected from students’ responses to questions such as “Please check the box next to the problems below which you have been worried about recently”. The content of worry included school, health, family, and interpersonal relationship concerns. They could also write down anything that was not included in a given option. Students were able to check more than one problem if appropriate. The total number of worries that the students provided were included in the final analysis.

In the present study, we refer to ‘mentor’ as the person who can be consulted about a problems a student has. Information about the number of mentors was collected from the student’s responses to questions such as “Do you have any friends who you can consult about the problems you have?” The list included parents, friends, senior students, professors and no one. Additionally, they could write down anyone who was not included in a given option. The students were able to choose more than one option. The total number of mentors that the students checked was calculated and used in the final analysis. Information about the number of campus social networks was obtained from the students’ responses to questions such as “How did you get to know your close friends?” The list included alumni associations, clubs, religious groups, dormitory roommates, classmates, and I don’t have a close friend. Students were also allowed to write down any social networks they were a member of in a blank box. The total number of social networks was included in the final analysis. With an open question, daily sleep duration was measured from students’ reports in terms of average duration spent sleep daily such as “average sleeping hours: around ____hours ____ minutes”. Using a similar method (open question), daily amount of video gaming (“average hours video game per day: ___hours”), and daily amount of internet searching on computer or smartphone (“average hours searching the internet on computer or smartphone: ____hours”) was collected from the students’ reports in terms of average duration spent daily asleep, video game playing or internet searching.

### Statistical analyses

Correlational analyses of the seven variables (the number of worries, mentors, campus social networks, daily amount of video game playing, daily amount of internet searching, daily sleep duration, depression) were performed using SAS Software (version 9.3; SAS Institute, Cary, NC). Moderated mediation analyses were executed on the SAS version 9.3 macro utilizing PROCESS (model 59) provided by Hayes [43]. PROCESS was performed using one independent variable (worry), three mediators (daily hours spent video game playing, daily hours searching the /internet on computer or smartphone, daily sleep duration), one moderator (mentors), and one dependent variable (depression). The same PROCESS was performed using another moderator (campus social networks). Additionally, the same analysis, aiming to examine the significance of one moderator (e.g., mentors) in a proposed model while controlling for the other moderator (e.g., campus social networks), were repeated. Finally, the first eigenvalue simultaneously obtained from two moderators through a principle component analysis was used as a moderator. Gender (male/female) and affiliation (undergraduate/graduate) were treated as covariates. The number of bootstrap samples for bias corrected bootstrap confidence intervals was 10,000. The normality of the data for each variable was checked using skewness, kurtosis values, visual inspection of histograms and box plots. Skewness and kurtosis has been reported in Table 1. To examine a moderated mediation analysis [43], the following conditions were considered: 1) if the effect of the independent variable on the mediator depends on the moderator, then the effect of mediator on the dependent variable must be significant, or if the effect of the mediator on the dependent variable depends on the moderator, then the effect of the independent variable on the mediator should be significant; 2) the conditional indirect effect of the independent variable on the dependent variable via the mediator depends on the presence of a certain range of the moderator. To demonstrate moderated mediation, the second condition is essential.

**Table 1:** Mean, Standard deviation, Correlation between main variables (N =6068)

	**Variables**	**1**	**2**	**3**	**4**	**5**	**6**	**7**
1	Depressive symptoms	-						
2	Number of worries	.36^***^	-					
3	Mentors	-.07^***^	.23^***^	-				
4	Campus social networks	-.07^***^	.11^***^	.22^***^	-			
5	Daily hours spent video gaming	.04^**^	.07^***^	-.03^*^	.09^***^	-		
6	Daily hours spent internet searching	.12^***^	.09^***^	.02	.06^***^	.08^***^	-	
7	Daily sleep duration	-.07^***^	-.06^***^	-.05^***^	-.02	.14^***^	.03^*^	-
	Mean	4.51	1.72	1.87	1.92	0.55	1.74	7.02
	Standard deviation	5.20	1.61	1.37	0.89	0.90	1.20	1.08
	Range: Maximum ~ Minimum	0 ~ 49	0 ~ 10	0 ~ 5	0 ~ 5	0 ~ 9	0 ~ 12	1 ~ 12
	Skewness	2.00	1.02	0.19	0.46	2.30	1.70	0.04
	Kurtosis	6.38	1.06	-0.81	-0.47	7.87	6.50	1.07

Note. ^***^p < 0.001, ^**^p < 0.01, ^*^p < 0.05.

## Results

### Preliminary correlation analyses

Table 1 presents means, standard deviations, ranges of values, and the correlations between the seven variables. The results showed that depression was positively associated with the number of worries and daily amount of internet searching. The relationship between depression and daily hours spent video gaming appeared to be significant, but the correlation coefficient indicated that the effect sizes between the two variables were very small. Number of mentors, number of campus social networks, and daily sleep duration were inversely correlated with depression. The number of worries was positively associated with daily hours spent video gaming and daily amount of internet searching, and negatively related to daily sleep duration. The number of mentors was inversely related to daily hours spent video gaming and daily sleep duration. The daily hours spent video gaming was positively correlated with daily sleep duration. However, although *p* values between the variables were significant, it should be noted that the effect sizes between the variables appeared to be very weak.

### Mediation analyses

Mediation: the results of the mediation analysis showed that the number of worries was significantly associated with the amount of time students spent playing video gaming, and it was inversely related to daily sleep duration. Daily hours spent video gaming had a positive effect on depression, and daily sleep duration had a negative effect on depression (all ps < 0.001 in Table 2). The number of worries was also significantly associated with depression in both models (all ps < 0.001 in Table 2). These results reveal that the relationship between the number of worries and depression was partially mediated by daily hours spent video gaming and daily sleep duration, respectively. A bootstrapped 95% confidence interval (CI) confirmed that the indirect effects of daily hours spent video gaming and daily sleep duration in the relationship between the number of worries and depressive symptoms were significant.

**Table 2:** Mediation analysis (N = 6068)

**Variables**	**B**	***SE***	***t***	***p***
Number of worries → Daily hours spent video gaming	.03	.01	4.58	<.001
Number of worries → Daily sleep duration	-.03	.01	-3.40	<.001
Daily hours spent video gaming → Depressive symptoms	.35	.07	4.91	<.001
Daily sleep duration → Depressive symptoms	-.21	.06	-3.72	<.001
Number of worries → Depressive symptoms	1.21	.15	10.13	<.001
**Bootstrap**	***Effect***	***SE***	**LL 95% CI**	**UL 95% CI**
	**Bootstrap results for indirect effect**			
Daily hours spent video gaming	.0114	.0035	.0058	.0194
Daily sleep duration	.0064	.0030	.0017	.0131

### Moderated mediation analyses

Since model 59, as proposed by Hayes [43] and used in the present study, did not allow two moderators in the same model, separate analyses were performed using each moderator (number of mentors, number of campus social networks), respectively. When we performed a moderated mediation, daily hours spent video gaming and daily sleep duration, but not daily hours spent searching the internet, appeared to be a significant mediator. As such, the following analysis dropped daily hours spent searching the internet as a mediator. Table 3 shows the results of the moderated mediation analysis when using daily hours spent video gaming and daily sleep duration as the mediators, and number of mentors as a moderator, in the relationship between the number of worries and depression. Table 4 presents the results of the moderated mediation analysis when entered daily hours spent video gaming and daily sleep duration were entered as the mediators, and the number of campus social networks was entered as a moderator, in the association between number of worries and depression.

**Table 3:** Moderated mediation analysis when using number of mentors as a moderator (N =6068)

***Mediator variable model***						
	**Outcome variable: Daily hours spent video gaming**			**Outcome variable: Daily sleep duration**		
	**B ( *SE *)**	***t***	***p***	**B ( *SE *)**	***t***	***p***
Number of Worries	.04 (.01)	5.20	<.001	-.02 (.01)	-2.74	.006
Number of Mentors	-.03 (.01)	-3.68	.001	-.03 (.01)	-2.51	.01
Interaction 1	-.01 (.00)	-2.29	.02	-.00 (.01)	-.01	*NS*
***Dependent variable model***						
	**Outcome variable: depressive symptoms**					
	**B ( *SE *)**		***t***		***p***	
Daily hours spent video gaming	.30 (.07)		4.19		<.001	
Daily sleep duration	-.23 (.06)		-4.10		<.001	
Number of Worries	1.36 (.04)		34.58		<.001	
Interaction 2	.02 (.05)		.30		*NS*	
Interaction 3	-.06 (.04)		-1.39		*NS*	
Number of Mentors	-.74 (.05)		-16.09		<.001	
Interaction 4	-.33 (.03)		-12.48		<.001	
***Conditional indirect effect at specific levels of the moderator***						
**Mediator**	**Moderator: number of mentors**		**Indirect effect (SE)**		**LL 95% CI**	**UL 95% CI**
Daily hours spent video gaming	0 (10^th^ percentile)		.0156 (.0083)		.0022	.0352
	1 (25^th^ percentile)		.0134 (.0050)		.0053	.0252
	2 (50^th^ percentile)		.0108 (.0035)		.0052	.0191
	3 (75^th^ percentile)		.0078 (.0035)		.0026	.0166
	4 (90^th^ percentile)		.0045 (.0042)		-.0014	.0162

Notes. Interaction 1: number of worries x number of mentors; Interaction 2: daily hours spent video game playing x number of mentors; Interaction 3: Daily sleep duration x number of mentors; Interaction 4: number of worries x number of mentors.

**Table 4:** Moderated mediation analysis when using number of campus social networks as a moderator (N =6068)

***Mediator variable model***						
	**Outcome variable: Daily hours spent video gaming**			**Outcome variable: Daily sleep duration**		
	**B ( *SE *)**	***t***	***p***	**B ( *SE *)**	***t***	***p***
Number of Worries	.03 (.01)	4.62	<.001	-.03 (.01)	-3.46	.001
Number of campus social networks	.04 (.01)	3.14	.002	-.01 (.02)	-.75	*NS*
Interaction 1	-.03 (.01)	-3.40	.001	.01 (.01)	1.31	*NS*
***Dependent variable model***						
	**Outcome variable: depressive symptoms**					
	**B ( *SE *)**		***t***		***p***	
Daily hours spent video gaming	.35 (.07)		4.95		<.001	
Daily sleep duration	-.21 (.06)		-3.66		.001	
Number of Worries	1.23 (.04)		31.38		<.001	
Interaction 2	.11 (.08)		1.41		*NS*	
Interaction 3	.03 (.06)		.40		*NS*	
Number of campus social networks	-.50 (.07)		-6.91		<.001	
Interaction 4	-.21 (.04)		-5.00		<.001	
***Conditional indirect effect at specific levels of the moderator***						
**Mediator**	**Moderator: number of campus social networks**		**Indirect effect (SE)**		**LL 95% CI**	**UL 95% CI**
Daily hours spent video gaming	0.90 (10^th^ percentile)		.0170 (.0086)		.0024	.0363
	0.96 (25^th^ percentile)		.0140 (.0052)		.0052	.0263
	1.53 (50^th^ percentile)		.0108 (.0035)		.0053	.0193
	2.50 (75^th^ percentile)		.0076 (.0035)		-.0025	.0161
	2.75 (90^th^ percentile)		.0042 (.0041)		-.0015	.0165

Notes. Interaction 1: number of worries x number of campus social network; Interaction 2: daily hours spent video game playing x number of campus social network; Interaction 3: Daily sleep duration x number of campus social network; Interaction 4: number of worries x number of campus social network.

As the mediation results were already reported, here we directly report the interaction effects between the variables included in the proposed model. Results showed that the interaction between the number of worries and the number of mentors (interaction 1 in Table 2), and between the number of worries and the number of campus social networks (interaction 1 in Table 3), respectively, were significant for daily hours spent video gaming but not for daily sleep duration. This implies that the effect of worry on daily hours spent video gaming was moderated by the number of campus social networks as well as the number of mentors. However, the interactions between the number of mentors and daily hours spent video gaming and between the number of campus social networks and daily hours spent video gaming (Interaction 2 in Tables 3 and 4, respectively) were not significant. Also, the interactions between the number of mentors and daily sleep duration and between the number of campus social networks and daily sleep duration were not significant (Interaction 3 in Tables 3 and 4). The direct effect of the number of worries on depression appeared to be dependent on the number of mentors in both models (both ps < 0.001; 4 in Tables 3 and 4).

**Figure 2:**
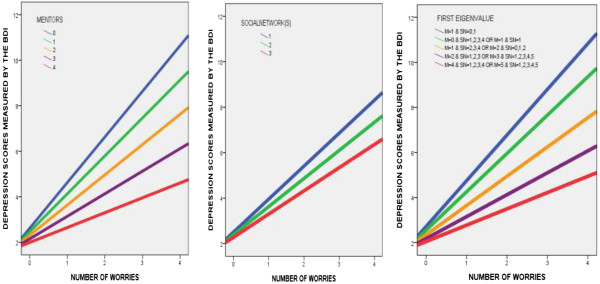
**The number of worries as a function of mentors, campus social networks, or both (first eigenvalue).** This interaction predicts daily duration of video gaming. The slope indicates that as the number of worries increases daily duration of video gaming also increases. However, the slope is increasingly reduced as the number of mentors, campus social network, or both increases. Note) M = Mentors; SN = Social Networks.

**Figure 3:**
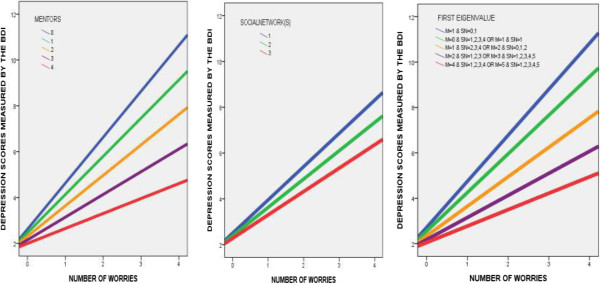
**The number of worries as a function of mentors, campus social networks, or both (first eigenvalue).** This interaction predicts the severity of depressive symptoms. The slope indicates that as the number of worries increases the severity of depressive symptoms also increases. However, the slope is increasingly reduced as the number of mentors, campus social network, or both increases. Note) M = Mentors; SN = Social Networks.

Conditional indirect effect: a bootstrapped 95% confidence interval (CI) confirmed that the indirect effects of daily sleep duration and daily hours spent video gaming in the relationship between the number of worries and depressive symptoms were significant (LLCI: 0.0018, ULCI: 0.0143 for daily sleep duration; LLCI: 0.0054, ULCI: 0.019 for daily hours spent game playing). Next, the indirect effect of number of worries on depression via daily hours spent video gaming differed depending on the range of number of mentors and campus social networks. Therefore, it was examined whether this indirect effect would be still significant through a bootstrapping method. The result shown in Table 3 confirmed that the conditional indirect effect of the number of worries on depression via daily hours spent video gaming differed depending on the range of the number of mentors. More specifically, this indirect effect was weaker in the high 10th percentile of number of mentors (Number of mentors > =4) whereas such a conditional indirect effect was significant in up to the 75th percentile of the number of mentors (A bootstrapped 95% CI did not include zero). These results indicate that the more worries a student has, the greater the amount of time the student spend playing video games. Therefore he or she is more susceptible to the development of depression. However, students with a higher number of mentors (high 10th percentile) can be protected from the risk of developing depression and spending time playing video games. Please see Figures 2 and 3. For campus social networks, the conditional indirect effect of the number of worries on depression via daily hours spent video gaming was significant in up to the 50^th^ percentile of the number of campus social networks; however, above the 75th percentile of social networks this effect was not significant (Table 4). These results imply that students who build a higher number of campus social networks are more protected from the risk of depression and video gaming. Please see Figures 2 and 3. As the direct effect of the number of worries on depression appeared to be moderated by the number of mentors (interaction 4 in Tables 3 and 4), it was examined whether this conditional direct effect would be significant. Unexpectedly, the conditional 95% CI for all ranges of moderators (number of mentors or campus social network) included zero. As such, the hypothesis that the relationship between the number of worries and depression was moderated by the number of mentors or the number of campus social networks was not supported.

**Figure 4:**
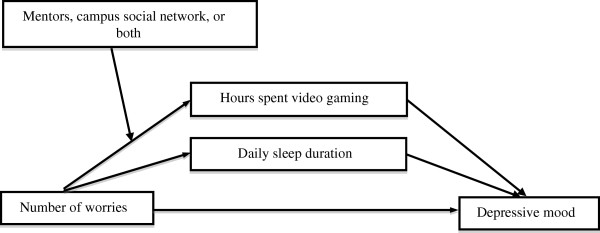
**The final moderated mediation model.** The link associating the number of worries and daily amount of video game with depression is moderated by mentors, campus social networks, or both.

Additional analyses: additional analyses were performed in an attempt to examine whether the effect of one moderator (i.e., mentors) in the proposed model of the present study would be still significant after controlling for another moderator (campus social networks). The results showed that the moderating effects of the number of mentors appeared to be significant after controlling for campus social networks. However, the effect of campus social networks was no longer significant after controlling for the number of mentors. Finally, the first eigenvalue obtained from data reduction using a PCA was also considered for an additional analysis. The first eigenvalue consisted of two factors, namely, the number of mentors and the number of campus social networks. As a result, similar patterns were observed; a conditional indirect effect of the upper 10th percentile of eigenvalue was not significant. That is, those who had at least four mentors and one campus social network simultaneously (the upper 10th percentile of eigenvalue) can be protected from video gaming and depression. The final moderated mediation model in the present study is shown in Figure 4.

Finally, given that the nature of cross sectional data, the possibility of a bidirectional association between the variables used in the present study cannot be excluded. For example, heightened depressive symptoms might lead to short sleep duration and increased video gaming, thereby causing a lot of worries. Therefore, when using depressive symptoms as an independent variable and number of worries as a dependent variable, whether the proposed moderated mediation pathways would still be significant was tested. The results showed that depressive symptoms significantly predicted number of worries (B = .11, p < .001) and video game use (B = .01, p = .004) but the rest of the pathways were not significant.

## Discussion

The main aim of the present study was to investigate a resilience model in the relationships between the number of worries, daily sleep duration, daily hours spent video gaming, the number of mentors, the number of campus social networks and depression, using a moderated mediation analysis. There were two key findings: firstly, daily hours spent video gaming and daily sleep duration partially mediated the association between the number of worries and depression. These results indicate that daily worries had both a direct and indirect effect on the severity of depression via video gaming and sleep duration. There has been a dearth of research investigating the specific mechanisms linking daily worries and depression severity. The present findings expand on the results of previous studies by demonstrating that video gaming and sleep duration mediate the association between daily worries and depression severity. Secondly, the mediating effect of daily hours spent video gaming on the relationship between the number of worries and depression was moderated by the number of mentors and the number of campus social networks, respectively. This finding highlights the importance of a social support system such as ‘mentorships’ or ‘befriending network’, in decreasing the tendency towards avoidant behaviors such as video gaming, and finally buffering susceptibility to depression.

Most studies have exclusively investigated the association between worry and anxiety. However, recent studies have increasingly noted that worry is equally related to both anxiety and depression [17,18,46], and our results are in line with such findings. Although depression and anxiety may have different cognitive schemata, pathological worry might render an individual susceptible to depression as well as anxiety [47].

Our results showed that worry also had an indirect effect on the severity of depression through daily video game playing. Although our cross-sectional data is limited in its ability to disentangle the causal links, these findings indicate that worry may confer a vulnerability to depression on university students through avoidant coping strategies such as excessive video game playing. One of the reasons that people play video games could be to escape from daily worries or concerns [48]. Indeed, in some case studies with video game players, Wood [31] found that the core impetus for excessive video game playing was to avoid real life problems, rather than an addiction to the video game itself. The present findings were the first to demonstrate that video game playing might act as an avoidant coping strategy for daily worries. It is likely that students who face a higher number of worries may spend a longer time playing video games in an attempt to avoid their worries, which might engender short sleep duration, irregular eating habits, time-management failures, and isolation from relationships, thereby leading to depression [49].

The result that worries also indirectly affect depression through shortened sleep duration is consistent with the previous findings that worry is highly associated with poor sleep, which in turn precipitates insomnia [19,20], and that short sleep duration contributes to the development of depression [21,50,51]. Short sleep duration or insomnia is commonly observed in those who have a tendency to have high levels of worries, intrusive thoughts, or a “racing mind” [19]. Based on previous research, it is possible that a lot of worries might fuel pre-sleep cognitive activity, which contributes to short sleep duration, and thereby depression. The present study provides additional evidence that worry is linked to depression through short sleep duration.

Interestingly, our findings indicate that not all students with a greater number of worries are immersed in playing video games, thereby experiencing depression. Some of them, who seek to a greater number of mentors (at least 4), or a higher number of campus social networks to build close relationships (at least 2), or have at least 4 mentors and 1 campus social network simultaneously, appeared to be protected from excessive video game playing and depression. In other words, when facing a greater number of worries, students who are active in seeking to mentors and campus social networks might be protected from excessive internet video game playing and, in turn, depression, whereas those who do not seek mentors or campus social networks might be more susceptible to depression through excessive video game playing. This indicates that not all students who display a higher number of worries are at a high risk of developing depression. As shown in Figures 2 and 3, ten percent of students who had at least 4 mentors without any campus social networks, or 25% of students who had at least 2 social networks without any mentor could overcome their risk of depression, indicating the importance of social support systems as an environmental resilience factor. A similar pattern was also observed in 10% of students who had at least 4 mentors and 1 campus social network simultaneously. The finding that the positive impact of mentors or campus social networks did not affect the link between video game playing and depression indicates the importance of early intervention through the promotion of communicative social networks before students use an avoidant strategy, such as video game playing as a means to avoid their worries.

### Limitations

The present study has several limitations: firstly, the design of the present study was cross-sectional, therefore, the results should be interpreted cautiously. Future study is warranted to clarify the causal links between the variables using an experimental or longitudinal design. Secondly, although the sample included in the current study was large (N = 6068), since the data was collected in one top-ranked, highly competitive national university, the generalizability of the current model should be replicated in a future study. Thirdly, the present study relied on self-report measures; including objective measures such as standardized interviews or observation would provide more accurate information. Also, the measures used in the current study assessed the quantity of worries, social networks and mentors. The sleep duration was also quantified. A useful recommendation for future research would be to investigate whether including the quality or severity of the worries, social networks, mentors and sleep duration would yield similar results. Finally, the current study found partial mediation of two potential mediators in the relationship between the number of worries and depression, which suggests that there would be other variables which mediate the association between the number of worries and depression. As such, future studies should include more mediators to provide a better understanding of the relationship between the number of worries and depression. Finally, the number of male and female students was disproportionate. For this reason, a future research with a large number of female participants is needed to determine whether the results of the present study can be generalized to female students.

## Conclusions

Despite the limitations of the present study, this is the first investigation of the potential moderated mediators of the association between worries and depression in university students. Our findings contribute to a better understanding of the specific interactive mechanisms underlying excessive worries and depression in university students. Based on our findings, actively utilizing campus social support systems such as ‘mentorships’ between seniors and juniors or between students and personal tutors or ‘befriending” social networks via online (e.g., Facebook) and offline (campus social club) might contribute to ameliorate the severity of depression in university students and decrease the economic costs of interventions for depression. The present study provides a useful theoretical and methodological approach regarding which factors should be targeted for the prevention of depression and who should be identified as being at high risk.

## Competing interests

The authors declare that they have no competing interests.

## Authors’ contributions

BS conceived the study and collected the data. JS performed the statistical analysis. JS and BS interpreted the results. JS reviewed the literature and wrote the manuscript. BS revised the manuscript. JS and BS approved the final version for publication. Both authors read and approved the final manuscript.

## Pre-publication history

The pre-publication history for this paper can be accessed here:

http://www.biomedcentral.com/1471-2458/14/426/prepub
